# Supplementary figures and images for: Enhancing HIV positivity yield in southern Mozambique: The effect of a Ministry of Health training module in targeted provider-initiated testing and counselling
Source: PLoS One. 2024 May 23;19(5):e0303063. doi: 10.1371/journal.pone.0303063 (PMC11115277; doi:10.1371/journal.pone.0303063)

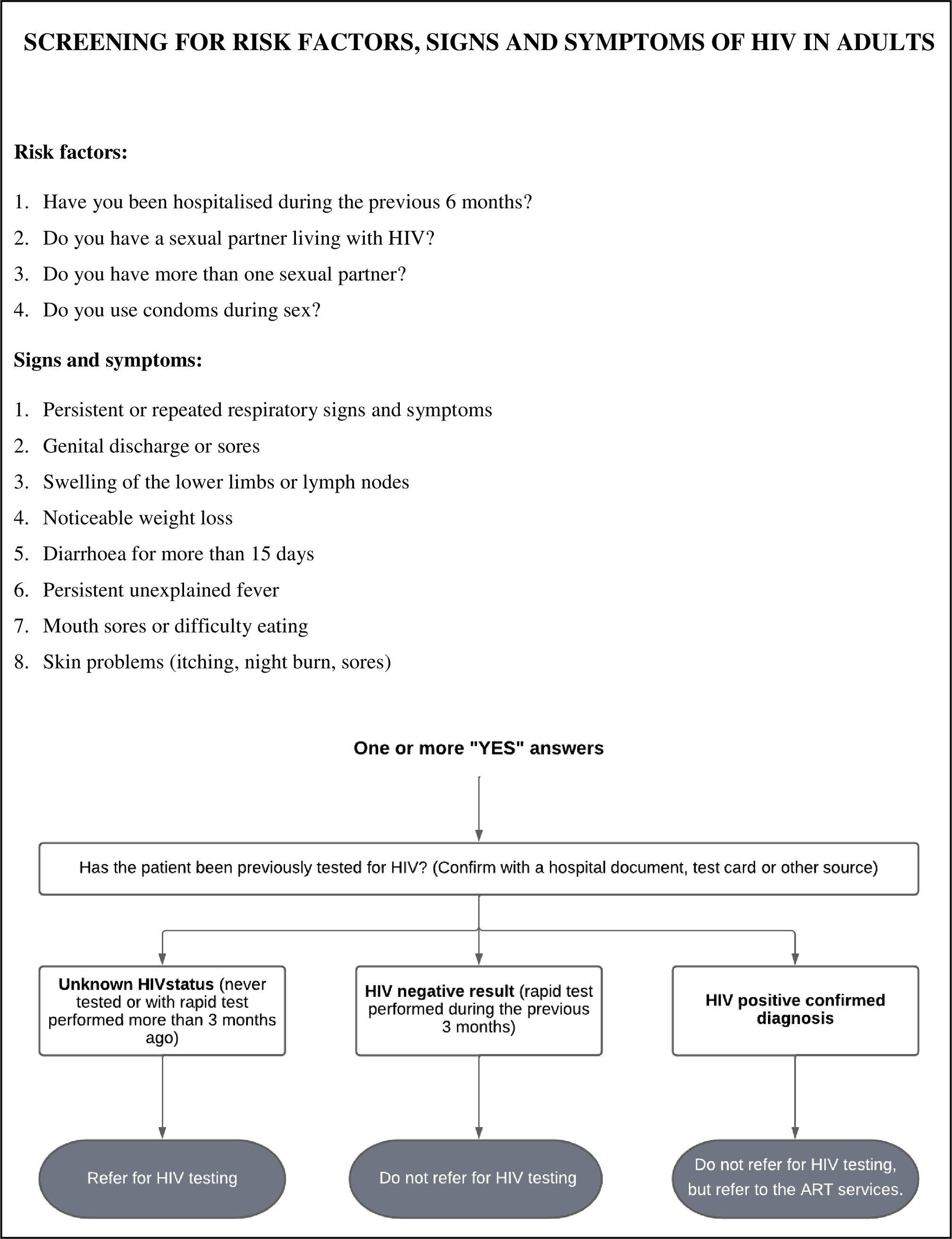

Supplement: S1 Fig — Figure adapted from the Differentiated Services Delivery Models Guidelines by the Mozambican MoH, 2018. Abbreviations: ART: antiretroviral therapy, PITC: provider-initiated testing and counselling. (TIF) [file pone.0303063.s001.tif]
