# Supplementary material for: Enhancing HIV positivity yield in southern Mozambique: The effect of a Ministry of Health training module in targeted provider-initiated testing and counselling
Source: PLoS One. 2024 May 23;19(5):e0303063. doi: 10.1371/journal.pone.0303063 (PMC11115277; doi:10.1371/journal.pone.0303063)
Supplement: Attachment — Submitted filename: Response to Reviewers.pdf [file pone.0303063.s003.pdf]

ISGlobal
Barcelona Institute for Global Health
info@isglobal.org
www.isglobal.org
Campus Clínic
Rosselló, 132
08036 Barcelona, España
T +34 93 227 1806
Campus Mar
Doctor Aiguader, 88
08003 Barcelona, España
T +34 93 214 7300

Barcelona, 10th January 2024

Re: Response to the editorial team and reviewers - PONE-D-23-28399

Dear editorial team and reviewers,

We would like to express our gratitude to the editorial team and reviewers for their thorough review of the manuscript. We have carefully read through and considered all comments and have found them very useful. We have revised the manuscript incorporating the revisions and have provided clean and tracked file versions of the revised manuscript, along with this detailed response to the comments.

Response to editor

Journal requirements:
When submitting your revision, we need you to address these additional requirements.

1. Please ensure that your manuscript meets PLOS ONE's style requirements, including those for file naming. The PLOS ONE style templates can be found at https://journals.plos.org/plosone/s/file?id=wjVg/PLOSOne_formatting_sample_main_body.pdf and https://journals.plos.org/plosone/s/file?id=ba62/PLOSOne_formatting_sample_title_authors_affiliations.pdf

RE: We appreciate the feedback regarding the adherence to PLOS ONE style requirements. After carefully reviewing the provided PLOS ONE style templates for manuscript formatting, we have meticulously aligned our manuscript with these guidelines. Our revisions involve adding level 1 and level 2 headings, applying

Fundación número 2634. Registro de Fundaciones Generalitat de Catalunya. NIF: G-65341695

EXCELENCIA SEVERO OCHOA
Institució CERCA Centres de Recerca de Catalunya
A partnership of:
"la Caixa" Foundation
CLÍNIC BARCELONA Hospital Universitari
UNIVERSITAT DE BARCELONA
Generalitat de Catalunya
GOBIERNO DE ESPAÑA
Parc de Salut MAR Barcelona
upf. Universitat Pompeu Fabra Barcelona
Ajuntament de Barcelona

indentation in each paragraph, including supplementary file legends in the Supporting information section of the main manuscript, and comprehensive revising file naming.

2. Please provide additional details regarding participant consent. In the ethics statement in the Methods and online submission information, please ensure that you have specified (1) whether consent was informed and (2) what type you obtained (for instance, written or verbal, and if verbal, how it was documented and witnessed). If your study included minors, state whether you obtained consent from parents or guardians. If the need for consent was waived by the ethics committee, please include this information.

If you are reporting a retrospective study of medical records or archived samples, please ensure that you have discussed whether all data were fully anonymized before you accessed them and/or whether the IRB or ethics committee waived the requirement for informed consent. If patients provided informed written consent to have data from their medical records used in research, please include this information.

RE: Thank you for the feedback on the Ethics statement and participant consent details. We have revised and updated the Ethics statement in the Methods section (line 230), addressing the approach for both observation and routine study phases: "All study participants, both healthcare providers and individuals presenting at the healthcare facilities during the observation phases of the study, completed written IC. For participants aged 15-18 years, a parent/legal representative's additional consent was necessary. Regarding the routine phases, as we extracted de-identified aggregated data, informed consent was not required". Should there be a necessity for further clarification or additional information on participant consent, we are prepared to provide any necessary details as guided by your instructions.

3. Thank you for stating the following in the Acknowledgments Section of your manuscript: "We acknowledge support from the grant CEX2018-000806-S funded by MCIN/AEI/ 10.13039/501100011033, and support from the Generalitat de Catalunya

Fundación número 2634. Registro de Fundaciones Generalitat de Catalunya. NIF: G-65341695

through the CERCA Programme. The authors gratefully acknowledge the staff at Centro de Investigação em Saúde de Manhiça, in the Manhiça District, Mozambique, who worked to collect and manage the data, the Ministry of Health of Mozambique, our research team, collaborators, and especially all communities and participants involved."

We note that you have provided funding information that is not currently declared in your Funding Statement. However, funding information should not appear in the Acknowledgments section or other areas of your manuscript. We will only publish funding information present in the Funding Statement section of the online submission form.

Please remove any funding-related text from the manuscript and let us know how you would like to update your Funding Statement. Currently, your Funding Statement reads as follows: "This publication has been supported by the President's Emergency Plan for AIDS Relief (PEPFAR) through the Centers for Disease Control and Prevention (CDC) under the terms of NU2GGH002092, the Severo Ochoa predoctoral fellowship by the Barcelona Institute of Global Health (ISGlobal) to ASL, the predoctoral fellowship from the Secretariat of Universities and Research, Ministry of Enterprise and Knowledge of the Government of Catalonia and cofounded by European Social Fund to ASL and SFL, and the European Respiratory Society (ERS) and the European Union (EU)'s H2020 research and innovation programme under the Marie Sklodowska-Curie grant agreement [847462] to ELV (This publication reflects only the author's view. The ERS, Research Executive Agency and EU are not responsible for any use that may be made of the information it contains). SW, employed by CDC participated in the conceptualization, study design and manuscript revision. For the remaining authors none were declared.
The findings and conclusions in this report are those of the author(s) and do not necessarily represent the official position of the funding agencies."

Please include your amended statements within your cover letter; we will change the online submission form on your behalf.

Fundación número 2634. Registro de Fundaciones Generalitat de Catalunya. NIF: G-65341695

RE: We appreciate the guidance on the appropriate placement of funding information in our manuscript. The funding acknowledgement mentioned in this sentence "We acknowledge support from the grant CEX2018-000806-S funded by MCIN/AEI/ 10.13039/501100011033, and support from the Generalitat de Catalunya through the CERCA Programme", is not directly associated with the funding for this study. Our institution requires us to include this information in the acknowledgements. However, as requested by the editor, we have removed it from the manuscript and reallocated it at the end of the funding statement.

Therefore, the final funding statement will read as follows: "This publication has been supported by the President's Emergency Plan for AIDS Relief (PEPFAR) through the Centers for Disease Control and Prevention (CDC) under the terms of NU2GGH002092, the Severo Ochoa predoctoral fellowship by the Barcelona Institute of Global Health (ISGlobal) to ASL, the predoctoral fellowship from the Secretariat of Universities and Research, Ministry of Enterprise and Knowledge of the Government of Catalonia and cofounded by European Social Fund to ASL and SFL, and the European Respiratory Society (ERS) and the European Union (EU)'s H2020 research and innovation programme under the Marie Sklodowska-Curie grant agreement [847462] to ELV (This publication reflects only the author's view. The ERS, Research Executive Agency and EU are not responsible for any use that may be made of the information it contains). SW, employed by CDC participated in the conceptualization, study design and manuscript revision. For the remaining authors none were declared. The findings and conclusions in this report are those of the author(s) and do not necessarily represent the official position of the funding agencies. Lastly, we acknowledge support from the grant CEX2018-000806-S funded by MCIN/AEI/ 10.13039/501100011033, and support from the Generalitat de Catalunya through the CERCA Programme."

4. We note that you have indicated that data from this study are available upon request. PLOS only allows data to be available upon request if there are legal or ethical restrictions on sharing data publicly. For more information on unacceptable data access

Fundación número 2634. Registro de Fundaciones Generalitat de Catalunya. NIF: G-65341695

restrictions, please see http://journals.plos.org/plosone/s/data-availability#loc-unacceptable-data-access-restrictions.

In your revised cover letter, please address the following prompts:

a) If there are ethical or legal restrictions on sharing a de-identified data set, please explain them in detail (e.g., data contain potentially sensitive information, data are owned by a third-party organization, etc.) and who has imposed them (e.g., an ethics committee). Please also provide contact information for a data access committee, ethics committee, or other institutional body to which data requests may be sent.

b) If there are no restrictions, please upload the minimal anonymized data set necessary to replicate your study findings as either Supporting Information files or to a stable, public repository and provide us with the relevant URLs, DOIs, or accession numbers. For a list of acceptable repositories, please see http://journals.plos.org/plosone/s/data-availability#loc-recommended-repositories.

We will update your Data Availability statement on your behalf to reflect the information you provide.

RE: Thank you for bringing up the concern regarding the data availability statement. In response to your guidance, we propose the following updates: "The data cannot be publicly shared due to ethical restrictions. Data contain potentially sensitive information, and the national ethics committee (CNBS) does not authorize data sharing without a protocol request specifying the objectives and the researchers who will have access to the data. The datasets generated and/or analysed during the current study are available upon request (contact via llorenc.quinto@isglobal) for researchers who meet the criteria for accessing confidential data."

Fundación número 2634. Registro de Fundaciones Generalitat de Catalunya. NIF: G-65341695

Response to reviewers

Reviewer #1:

Review Comments to the Author

The study brings a very important message in reaching the last mile of the first 95% UNAIDS target, particularly for Mozambique where the HIV burden is still high. However, as a general comment I think the generalizability of the study results is compromised first because the study was limited to a single geographical area in Mozambique (Mozambique is a sizeable country with a lot of diversity, even within the health sector). Second, the study did not include a control group which would have allowed for a more meaningful comparison. Finally, I think if the study had been conducted over a long period of time, one could have been able to assess long term trends and more accurate predictions.

However, I applaud the investigators as despite the limitations the study raises some important points for programs to consider: 1) if the current algorithms are still adequate for the last mile, 2) the current training is it enough, frequency, quality and 3) application of evidence based strategies. For this I believe this article is very important.

RE: Thank you for the comprehensive overview and recognizing the study's importance in shaping HIV programs toward achieving the last mile of the first 95 UNAIDS target. We acknowledge that the absence of a control group may impact the ability of draw causal inferences. This limitation has been appropriately addressed and discussed in the manuscript's Discussion section at line 482. Regarding the duration of the study, as our analysis did not focus on trends, this factor did not impact our findings. However, as this is a first step, we recognize the importance of conducting a longer-term study to analyse future trends.

Fundación número 2634. Registro de Fundaciones Generalitat de Catalunya. NIF: G-65341695

Concerning the study's restriction to a rural setting, we acknowledge the reviewer's point about potential limitations in generalizing the results to urban areas. However, as elaborated further in a subsequent comment, our intentional focus was on a rural area marked by a significant HIV burden and constrained resources

Some few comments:

- *Was the training online or was it in person, what was the duration of the training and how many health professionals were trained?*

RE: Thank you for the comment. The training sessions covering signs, symptoms, and risk factors for HIV (targeted PITC) were conducted in person by the HIV testing and counselling reference group from the Mozambican Ministry of Health at the Manhiça District hospital. These details have been added in line 183 of the manuscript. The training spanned one week, from the 13th to the 19th of May (refer to Figure 1 for clarification). The 19 healthcare professionals who participated in the study were the same individuals who attended this training module, as mentioned in lines 149 and 235, having agreed to take part in both study phases and the training.

- *The study was conducted in a rural area in Maputo province. Could the authors explain the choice of the health facilities? Why were some urban health facilities in Maputo not included?*

RE: Thank you for the insightful comment. The authors chose to focus the study on a semi-rural area due to its high HIV prevalence and the strain on its healthcare system, which operates with limited resources. These details have been included in the Methods section at line 134. Our aim was to contribute insights into rural areas, often overshadowed by urban areas, despite their crucial role in the "last mile" efforts targeting harder-to-reach populations. We have made specific modifications throughout the manuscript (lines 51, 71, 123, 385, 431 and 501) to emphasize that our study setting and findings are centred on rural areas. Additionally, our team has established a longstanding

Fundación número 2634. Registro de Fundaciones Generalitat de Catalunya. NIF: G-65341695

collaboration with the local HIV program implementer, Fundação Ariel, which significantly supported our research efforts in this area. The selection of the healthcare facilities within the study area was based on patient volume, including the four highest-volume facilities in the district (line 132).

Acknowledging Mozambique's diverse healthcare landscape, as highlighted by the reviewer in the overview, we recognize that our specific site selection might limit generalizability to urban areas. However, given our study’s focus on rural challenges, the authors do not consider this a limitation and believe our findings hold potential applicability to other rural areas facing similar challenges of a high HIV burden and constrained resources.

- *Could the authors please specify the professional level of the health professionals in the triage and emergency department areas? Could this have influenced in the results for example if some facilities had doctors vs nurses in triage?*

RE: We appreciate the comment. All 19 healthcare providers included in the study were medical assistants (line 235), who, in Mozambique, undergo four years training in basic medical care, allowing them to prescribe medicines and support minor surgeries. This uniform professional level minimised the potential influence of professional level diversity in our analysis. However, as the reviewer infers, if the training were applied to healthcare workers with variations in professional levels, the impact of training on HIV testing yield could differ based on the pre-training experience and background of individuals. We have included this important observation in the Discussion section at line 493. It is important to note that HIV testing referral tasks in Mozambique are primarily carried out by medical assistants and nurses.

- *Line 417 - included references twice, please delete one*

Fundación número 2634. Registro de Fundaciones Generalitat de Catalunya. NIF: G-65341695

RE: Thank you for highlighting the error. We have now removed the duplicated references as per your observation.

Reviewer #2:

Review Comments to the Author

Global overview

This is an interesting study measuring the effectiveness of a training module to improve the yield of PITC in triage and emergency departments using a pre-post study design. While acknowledging the significance of the study, I find that the manuscript lacks focus and requires a more cautious interpretation of some results.

Since their main objective is to enhance "positivity yield" (i.e., the sensitivity of the screening algorithm), I do believe they should consider the volume of new positive cases in the assessment. I understand that we want to improve the yield to reduce time burden and cost of doing systematic test but this should not be at the expense of the volume of newly diagnosed individuals (e.g., for a same denominator, it is better to have a yield of 5% if it leads to 100 new cases of HIV positivity rather than a yield of 10% and only 10 new cases of HIV positivity). Usually, this information would be given by sensibility (which would have provided the number of true HIV negative excluded), but the current study design does not allow to infer on that aspect. Thus, I believe this is important to provide alongside those yields, the number of new cases with the number of eligible population (e.g., patient seen in emergency services).

The authors have included a secondary objective which aims to improve the current PITC algorithm. I am not comfortable with that analysis as I have some doubts about the generalization of their results. There is not much description for the methods of that secondary objective especially on how the 509 positives tests have been included in the analysis (during the intervention, before or both?), and the fact that this population has

Fundación número 2634. Registro de Fundaciones Generalitat de Catalunya. NIF: G-65341695

been already selected through provider perceptions or PITC algorithm can potentially create a selection bias. I would suggest removing that part to refocus on the main objective of the paper or add sufficient information to be able to assess those results and be more cautious on the interpretation of the results.

RE: Thank you for the comprehensive feedback, recognizing the significance of the study. We have made concerted efforts to address reviewer' suggestions and refine our focus.

Our primary focus on yield aimed to assess the operational impact of the training module in targeted PITC (line 104). We acknowledge, in alignment with the reviewer's comment, that presenting HIV positivity yield as a proportion might not offer a complete understanding. To provide a more comprehensive view, we have included both the numerator (total positive individuals among those referred and tested) and denominator (total individuals referred and tested) when reporting yields (lines 58-61, 302-306, 315-320 and 322-328). This approach allows for a clearer understanding of the absolute number of positive individuals identified, even if a higher yield results in a lower count of positive cases. While testing a larger population and identifying more positives is the ideal scenario in well-resourced settings, the reality of resource constraints in contexts such as the rural area of our study presents challenges in offering universal testing. Therefore, the Ministry of Mozambique seeks strategies, such as targeted PITC, to optimize available resources —a shared challenge in other regions of southern Africa. We have expanded on this aspect in the Discussion section at line 424.

Regarding sensitivity estimation, it is crucial to note that during both observation phases, healthcare providers referred individuals for HIV testing in a manner closer to universal testing than targeted PITC, potentially influenced by the study (Hawthorne effect) (aspect explained in the Discussion section at line 397). As a result, the sensitivity would reflect the performance of healthcare providers through universal testing, not targeted PITC. For reviewer presentation, we estimated sensitivity using the values depicted in Figure 2. Sensitivity was estimated as the number of positives detected through universal testing

Fundación número 2634. Registro de Fundaciones Generalitat de Catalunya. NIF: G-65341695

within the total positives. In the pre-training observation phase, sensitivity was 92% (269/291); 95%CI: 89-95%, and in the post-training observation phase, it reached 95% (207/218); 95%CI: 91-97%. For the routine phases, reliance on real-world daily testing data without implementing universal testing made it unfeasible to estimate sensitivity due to the unknown total number of HIV positives. Considering these limitations, we opted not to include the sensitivity values in the manuscript.

We have also revised our manuscript to enhance clarity regarding the secondary objective (lines 47, 125 and 217), which aimed to evaluate factors associated with a positive HIV test, following similar methodologies used in other studies such as Abdul et al, 2023. This analysis involved all 509 individuals who tested positive during both observation phases (line 331). Throughout these phases, we collected data on sociodemographic characteristics, signs, symptoms and risk factors suggestive of HIV. These individuals represent all the positive individuals among those who visited the healthcare facilities during the observation phases. In our study, additional HIV study counsellors conducted tests for all individuals without a known HIV diagnosis, regardless of whether they were referred through PITC by healthcare providers (lines 196). This way, we minimised the risk of selection bias.

Lastly, we agree with the reviewer's point referring to the fact that our methodology did not directly evaluate the improvement of the current PITC algorithm. Consequently, we have rephrased the section where we discussed the optimisation of the current PITC algorithm to avoid any confusion regarding our study's objectives (line 367). We explicitly clarified that the 2.6% increase in new diagnoses by including new factors, and the 3.5% increase by removing the recent-HIV negative test exclusion criteria were observed within our study population (lines 65-68, 379, 392 and 456-470). The significance of these findings lies in their potential to guide further studies in evaluating the performance of the targeted PITC algorithm following these potential adaptations.

Title

Fundación número 2634. Registro de Fundaciones Generalitat de Catalunya. NIF: G-65341695

The title should accurately reflect the paper's focus on "positivity yield" rather than "HIV testing yield". This terminology should be corrected throughout their manuscript.

RE: Thank you for the suggestion. We agree that "HIV positivity yield" more accurately represents the focus of our paper. We have made the necessary adjustments, modifying the title and ensuring consistency with this term throughout the manuscript. To avoid confusion with the term "HIV test positivity" (secondary objective), we have adjusted it to "positive HIV test" throughout the manuscript.

Abstract

Since the paper is about the positivity yield in four specific periods (pre-post intervention during routine practice and pre-post intervention during the study period), I think the authors should focus more on that specific aspect and present yields with numerators and denominators for each of the 4 periods.

RE: Thank you for the input concerning the abstract. We have included the numerators and denominators of the yields for the four periods (line 59).

Background

While the background is informative, it lacks references on the effectiveness of targeted PITC. It misses some key references about the effectiveness of targeted PITC (see one suggested below among many other ones).

Leblanc J, Hejblum G, Costagliola D, Durand-Zaleski I, Lert F, de Truchis P, Verbeke G, Rousseau A, Piquet H, Simon F, Pateron D, Simon T, Crémieux AC; DICI-VIH (Dépistage Infirmier CIblé du VIH) Group. Targeted HIV Screening in Eight Emergency Departments: The DICI-VIH Cluster-Randomized Two-Period Crossover Trial. Ann Emerg Med. 2018 Jul;72(1):41-53.e9. doi: 10.1016/j.annemergmed.2017.09.011. Epub 2017 Oct 31. PMID: 29092761.

Fundación número 2634. Registro de Fundaciones Generalitat de Catalunya. NIF: G-65341695

RE: Thank you for the suggestion. We primarily focused on Sub-Saharan African-based references (Ref 9-10, line 113), and included WHO guidelines that compile substantial evidence guiding their recommendation for targeted PITC (Ref 11). The reviewer suggested an example reference of broader spectrum, which is valuable, and we have incorporated it accordingly (Ref 12).

Methods

A description of the control period, especially how PITC was implemented before the intervention, would enhance the understanding of the study design.

RE: Thank you for the feedback. Our study adopted a single-group pre-post design to evaluate the impact of a new training module in targeted PITC, serving as the intervention. The control period referenced by the reviewer corresponds to the pre-intervention period, encompassing the pre-training routine phase (1 November 2018-28 February 2019) and the pre-training observation phase (1 March-12 May 2019), as illustrated in Fig 1. During this period, healthcare providers followed the national targeted PITC algorithm for referring individuals for HIV testing, as shown in S1 Fig. However, they had not yet received the new training module. In summary, healthcare providers referred individuals for HIV testing through targeted PITC both before and after the intervention, with the new training module introduced only during the post-training phases. We have integrated these details into the Introduction (line 117) and the Methods section (lines131 and 162) for better clarity.

Results

Figure 2 resolution is very low, I was not able to read it.

It would have been interesting to have a figure should the evolution of those positive yield per month or every two months to see the eventual fluctuation of positivity yield.

I am not certain to understand the drop of patients seen during the study period (n=7,102, ~1776 patients seen per month) compared to the routine period (n=33,261, ~ 4,158 per month).

Fundación número 2634. Registro de Fundaciones Generalitat de Catalunya. NIF: G-65341695

RE: Thank you for highlighting the concerns in the Results section. We have updated Figure 2 to improve its resolution, ensuring better readability.

We appreciate the reviewer's interest in illustrating the monthly evolution of HIV test positivity. However, our study design was not tailored for time series analysis or trend evaluation. Due to the limited number of time points and the small sample size per point, the validity of conducting a valid time series analysis, such as an interrupted time series analysis, could be compromised. As previously mentioned to reviewer 1, our study marks an initial step, and we fully acknowledge the need for a longer-term study to analyse future trends. Additionally, considering the difficulty in interpreting the yield alone (it should be reported along with the numerator and denominator, as also suggested by the reviewer), we do not consider yield as an appropriate outcome for time series analysis.

Regarding the lower number of individuals observed during the observation phases compared to the routine phases, several factors might explain this difference:

- We excluded unaccompanied individuals under 18 and non-residents of Manhiça district (line 152). A prior study in the same area (Fuente-Soro et al, 2018*)* found that 36% of positive cases involved individuals not residing in the district, potentially seeking testing in other districts due to stigma.
- During the observation phases, we recruited individuals attending the healthcare facility from 8 AM to 3 PM, Monday to Friday. In contrast, for the routine phases, we extracted aggregated data from data collection books that included all individuals accessing healthcare facilities round the clock, including weekends and bank holidays. It was not feasible to disaggregate the data by day or time.
- Approximately 18% of healthcare providers across the four facilities declined participation, primarily because their usual working hours did not align with the study hours (from 8 AM to 3 PM) or because they occasionally worked in the triage/ED but it was not their regular duty (line 148).

Fundación número 2634. Registro de Fundaciones Generalitat de Catalunya. NIF: G-65341695

The new clarifications have been incorporated into the Results sections (line 238 and 286).

Discussion

The discussion is mainly focusing on improving the PITC algorithm which is not the main topic on the paper.

Since the intervention had no effect during the study period but was found effective between the pre and post routine period, I think a broader discussion about that aspect (e.g., why this intervention did not work in the intervention period? Were there any study contaminations?) should be more highlighted here with the potential implication of such results.

RE: Thank you for the input on the Discussion section. We have implemented the following changes to address the reviewer' suggestions:

- At line 397, we have elaborated on why there was not a rise in yield during the observation phases ("The study itself is likely to have influenced providers to refer in a manner closer to universal PITC than to targeted PITC, even before they received training, due to their awareness of the ongoing evaluation of the PITC performance. This is known as the Hawthorne effect, which has been described in several SSA healthcare settings. Additionally, resources such as the number of HIV counsellors were increased for study purposes, so providers knew that all individuals referred for testing during the observation phases would be tested, in contrast to routine conditions"). To improve clarity, we have added a sentence at line 406.
- We have included a paragraph at line 424, delving deeper into the yield as an operational indicator of the performance of targeted PITC. This addresses the reviewer's request in the global overview regarding this aspect.
- To streamline the discussion, we have removed the paragraph at line 473, consolidating the discussion about the factors associated with a positive HIV test (secondary objective) into a single paragraph at line 451.

Fundación número 2634. Registro de Fundaciones Generalitat de Catalunya. NIF: G-65341695

- Lastly, the conclusions (line 499) have been revised to accurately reflect the implications drawn from our study findings.

Thank you for considering this article for publication in your journal. I look forward to hearing from you soon.

Please accept my highest assurances.

Yours sincerely,

Anna Saura Lázaro

Anna Saura

ISGlobal, Hospital Clínic - Universitat de Barcelona, C/ Rosselló, 132, 5th 2nd. 08036. Barcelona, Spain. Phone: (+34) 652413532. Email: anna.saura@isglobal.org

Fundación número 2634. Registro de Fundaciones Generalitat de Catalunya. NIF: G-65341695
