# Supplementary material for: Enhancing HIV positivity yield in southern Mozambique: The effect of a Ministry of Health training module in targeted provider-initiated testing and counselling
Source: PLoS One. 2024 May 23;19(5):e0303063. doi: 10.1371/journal.pone.0303063 (PMC11115277; doi:10.1371/journal.pone.0303063)
Supplement: Attachment — Submitted filename: RESPON~1.pdf [file pone.0303063.s004.pdf]

ISGlobal

Barcelona
Institute for
Global Health

info@isglobal.org
www.isglobal.org

Campus Clínic
Rosselló, 132
08036 Barcelona, España
T +34 93 227 1806

Campus Mar
Doctor Aiguader, 88
08003 Barcelona, España
T +34 93 214 7300

**Barcelona, 16th April 2024**

**Re: Response to the editorial team and reviewers - PONE-D-23-28399R1**

Dear editorial team and reviewers,

We would like to express our gratitude to the editorial team and reviewers for their thorough review of the first round of manuscript revisions. We have carefully read through and considered all comments and have found them very useful. We have revised the manuscript incorporating the revisions and have provided clean and tracked file versions of the revised manuscript, along with this detailed response to the comments. Additionally, we would like to inform the editor that we have expanded the abstract by 39 word to incorporate the information requested by the reviewer. However, we remain open to reducing this new information, should the editor deem it necessary.

***Response to reviewers***

**Reviewer #1:**

*All questions were adequately addressed and the authors made significant improvement to the manuscript. It is much better and clearer.*

**Reviewer #3**

*This is an interesting paper on the impact of training of health care providers in a primary care seting in rural Mozambique on targeted provider initiated HIV testing and counselling (PITC) and assessing the impact on HIV positivity yield among those in care within the study period (e.g. consented participants in care before and after the study period) and in the wider timeframe of 4 months prior to and after completion of the study looking at aggregate routine care data.*

*This submission had already responded to some reviewers comments.*

*Overall the study design and conclusions are sound although the way the results are*

Fundación número 2634. Registro de Fundaciones Generalitat de Catalunya. NIF: G-65341695

*presented and summarised is rather confusing and difficult to follow and would suggest some edits to address this.*

*Major comments:*

*Abstract*

• *It would be helpful for the reader to clarify the primary versus secondary outcomes of interest in this study and signpost these results accordingly. For example, I assume the primary outcomes are those based on the study 'observation phases' (n=7102, main outcome is HIV positivity yield overall and by sex). Secondary objectives – focusing on outcomes in the 'routine data phases'? If so, in the results the authors should provide the N for both primary and secondary objectives, in the pre and post period, with the sex distribution and proportion recruited at triage for consistency, before reporting the yield. It would also be important to include data on the number/proportion in care targeted for HIV testing before/after the study period to show decline in numbers tested in the post training period.*

RE: Thank you for the comment. The primary objective of our study was to evaluate the effect of a new Ministry of Health training module in targeted PITC on HIV positivity yield, which we identified as our primary outcome. For this evaluation we used data from both the observation and routine phases, with the latter phase serving to control for potential observer bias (Methods section, line 151). We have clarified the primary objective and outcome in the Introduction section at line 121 and in the Methods section at line 206. Our secondary objective was to identify factors associated with a positive HIV test, with odds ratios from logistic regression models serving as the secondary outcome. The secondary objective is detailed in the Introduction section at line 123 and the secondary outcome has been further clarified in the Methods section at line 214. In the abstract, we have incorporated the statistical analysis used for the secondary objective at line 56 to enhance clarity. Additionally, acknowledging the importance of consistency, and following the reviewer's suggestion, we have now included in the abstract, at line 59, the number of participants (N), broken down by sex and department, for the routine phases. Lastly, the number of individuals in care referred for HIV testing, as suggested in the first round of revisions, was included as the denominator for the HIV positivity yield results.

Fundación número 2634. Registro de Fundaciones Generalitat de Catalunya. NIF: G-65341695

• *Also important to state clearly that the analyses on factors associated with positive HIV test presented here is from the study period and not the routine data phase as it follows immediately after reporting the yield of the routine data phase.*

RE: We appreciate the comment and agree with the reviewer's suggestion. Accordingly, we have clarified in the Abstract at line 56 that the analysis of factors associated with a positive HIV test was based on data from the observation phases.

*Methods and results*

• *In the methods it states at during the study period, all eligible patients in care were asked to consent to be part of this study prior to being assessed for PITC and all offered HIV counselling and testing, irrespective of PITC recommendations to assess how many new HIV diagnoses were missed by targeted PITC. However, the authors do not describe how these data are used and compared to the number of new diagnoses captured through targeted PITC in the statistical analysis section of the methods. This seems to be a key strength in the study design but does not really stand out as a key result?*

RE: Thank you for the feedback and our apologies for any confusion caused. The data highlighted by the reviewer are indeed crucial and presented in Figure 2. Following the suggestion in the first round of revisions, we improved the resolution of Figure 2, which contains essential information. However, it appears that it was not legible in the PDF generated by the submission system and may need to be downloaded directly from the system. To aid in the review, we have attached Figure 2 to this letter. These findings are also presented in the Results section at line 366: "Among all participants who tested positive for HIV, 72.0% (364 out of 509) were eligible for testing under the current targeted PITC algorithm. This leaves 145 HIV diagnoses that were missed by the existing algorithm". Additionally, per reviewer's advice, we have further detailed how these data were analysed in the statistical analysis in the Methods section at line 217.

Fundación número 2634. Registro de Fundaciones Generalitat de Catalunya. NIF: G-65341695

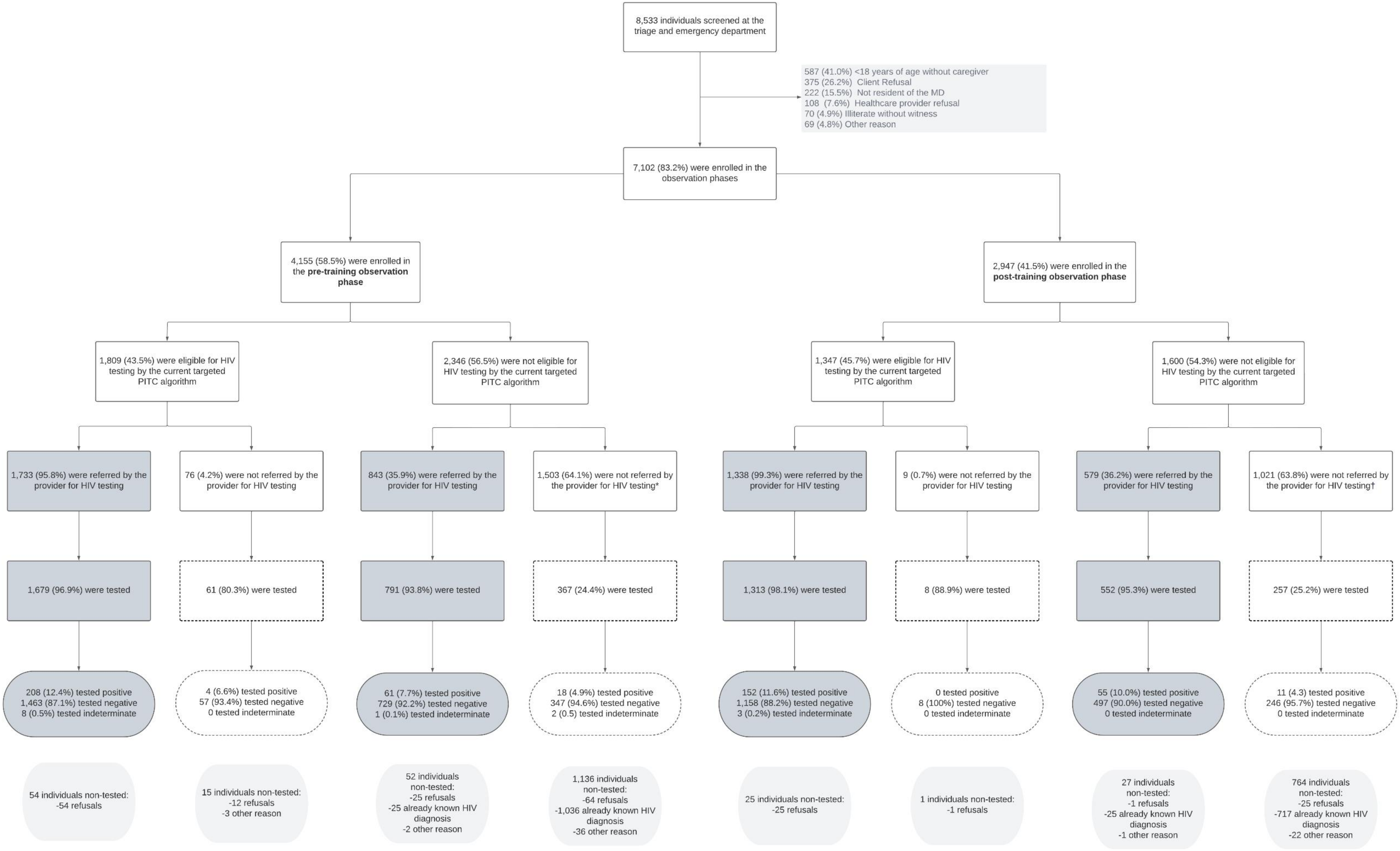
8,533 individuals screened at the triage and emergency department
587 (41.0%) <18 years of age without caregiver
375 (26.2%) Client Refusal
222 (15.5%) Not resident of the MD
108 (7.6%) Healthcare provider refusal
70 (4.9%) Illiterate without witness
69 (4.8%) Other reason
7,102 (83.2%) were enrolled in the observation phases
4,155 (58.5%) were enrolled in the pre-training observation phase
2,947 (41.5%) were enrolled in the post-training observation phase
1,809 (43.5%) were eligible for HIV testing by the current targeted PITC algorithm
2,346 (56.5%) were not eligible for HIV testing by the current targeted PITC algorithm
1,347 (45.7%) were eligible for HIV testing by the current targeted PITC algorithm
1,600 (54.3%) were not eligible for HIV testing by the current targeted PITC algorithm
1,733 (95.8%) were referred by the provider for HIV testing
76 (4.2%) were not referred by the provider for HIV testing
843 (35.9%) were referred by the provider for HIV testing
1,503 (64.1%) were not referred by the provider for HIV testing*
1,338 (99.3%) were referred by the provider for HIV testing
9 (0.7%) were not referred by the provider for HIV testing
579 (36.2%) were referred by the provider for HIV testing
1,021 (63.8%) were not referred by the provider for HIV testing†
1,679 (96.9%) were tested
61 (80.3%) were tested
791 (93.8%) were tested
367 (24.4%) were tested
1,313 (98.1%) were tested
8 (88.9%) were tested
552 (95.3%) were tested
257 (25.2%) were tested
208 (12.4%) tested positive
1,463 (87.1%) tested negative
8 (0.5%) tested indeterminate
4 (6.6%) tested positive
57 (93.4%) tested negative
0 tested indeterminate
61 (7.7%) tested positive
729 (92.2%) tested negative
1 (0.1%) tested indeterminate
18 (4.9%) tested positive
347 (94.6%) tested negative
2 (0.5) tested indeterminate
152 (11.6%) tested positive
1,158 (88.2%) tested negative
3 (0.2%) tested indeterminate
0 tested positive
8 (100%) tested negative
0 tested indeterminate
55 (10.0%) tested positive
497 (90.0%) tested negative
0 tested indeterminate
11 (4.3) tested positive
246 (95.7%) tested negative
0 tested indeterminate
54 individuals non-tested: -54 refusals
15 individuals non-tested: -12 refusals -3 other reason
52 individuals non-tested: -25 refusals -25 already known HIV diagnosis -2 other reason
1,136 individuals non-tested: -64 refusals -1,036 already known HIV diagnosis -36 other reason
25 individuals non-tested: -25 refusals
1 individuals non-tested: -1 refusals
27 individuals non-tested: -1 refusals -25 already known HIV diagnosis -1 other reason
764 individuals non-tested: -25 refusals -717 already known HIV diagnosis -22 other reason

*• Results line 256-258: you provide the proportion of study participants in the study observation period who meet the PITC algorithm criteria and the % not meeting the criteria but still referred for PITC. It would be interesting to see if many of those not meeting the criteria had characteristics consistent with those identified in the regression analysis as being associated with HIV positive result?*

RE: Thank you for the comment. In our analysis addressing the secondary objective—to identify factors associated with a positive HIV test—we included all tested participants, irrespective of whether they were referred for HIV testing by the healthcare provider through targeted PITC (Methods section, line 192). This approach allowed us to uncover additional factors that are not considered in the current targeted PITC algorithm but could potentially increase the detection of HIV diagnoses. Indeed, we identified several factors associated with a positive HIV test that, if included as eligibility criteria, could help identify additional HIV-positive individuals deemed non-eligible by the current targeted PITC algorithm. These exploratory findings are detailed in the Results section at line 373, with further clarification provided at lines 366 and 375.

*• Table 1 summarises the characteristics of all participants enrolled in the study n=7102, although only a subgroup were selected for PITC, and the characteristics of those selected are not described. It would be more informative to expand on this table 1A, and give also the characteristics of the following subgroups within each pre/post observation phase groups (i) those selected for PITC and met the criteria of the algorithm, (ii) those selected for PITC but not met criteria of algorithm, (iii) and those not referred for PITC and for all subgroups include the number testing HIV positive in the table.*

RE: Thank you for the suggestion. As previously mentioned, the primary objective of our study was to evaluate the HIV positivity yield before and after implementing a training module in targeted PITC. Accordingly, we stratified the data in Table 1 to compare clients' characteristics pre- and post-training phases, to ensure the comparability of groups. We consider that adding further stratification by additional subgroups might make the table's interpretation more challenging and not shed further light on our primary or secondary objectives. However, we acknowledge the reviewer's point regarding the importance of presenting the actual figures within each subgroup. And indeed, this is

Fundación número 2634. Registro de Fundaciones Generalitat de Catalunya. NIF: G-65341695

presented in Figure 2, which stratifies participants by their eligibility for HIV testing through targeted PITC and by healthcare providers referrals.

• *Results line 296-300 and Table 2: this summarises the total referred for PITC in the pre/post observation period and number tested positive. What is missing is the number of study participants not referred to PITC who were tested positive e.g. missed new diagnoses. Where are these data? Would be particularly interesting to see by department, especially emergency where yield appears highest? Also it should be clearly stated in the results and discussion that these yield ratios are unadjusted for potentially varying characteristics of clients over time.*

RE: Thank you for the comment. The data regarding study participants who were not referred for HIV testing through targeted PITC by healthcare providers but tested positive are shown in Figure 2, enclosed above for reference. Regarding stratification by department, it is important to note that all participants enrolled in the emergency department who tested positive during the observation phases, including both pre- and post-training phases (n=49, inferred from data from Tables 2B and 3), were referred for HIV testing through targeted PITC by healthcare providers. This referral pattern was significantly influenced by observer bias, the Hawthorne effect—a point thoroughly discussed in the manuscript. Additionally, the higher HIV prevalence and generally more severe conditions of patients attending the emergency department likely contributed to the high frequency of referrals for HIV testing by healthcare providers. This context helps explain the high HIV positivity yield observed in this department and why there was no increase in HIV positivity yield after the training module, given that all positive individuals were referred by the provider during both observation phases. Consequently, the specific question raised by the reviewer regarding missed diagnosis by the healthcare providers through targeted PITC in the emergency department cannot be addressed. Additionally, the issue of unadjusted yield ratios has been recognized and is discussed as a limitation in the Discussion section at line 459.

• *Results: page 36 first 2 paragraphs discusses the increase in yield in the triage department but not the emergency department. It is worth noting the very high yield in the observation period overall at >15% and trends suggesting a reduction in yield post training although not statistically significant, likely due to smaller samples. Here in particular it would be interesting to know how many diagnoses were missed by PITC?*

Fundación número 2634. Registro de Fundaciones Generalitat de Catalunya. NIF: G-65341695

RE: Thank you for the suggestion. As detailed in our previous response, all participants who tested positive for HIV in the emergency department during both pre- and post-training observation phases were referred for HIV testing by healthcare providers. This referral pattern was primarily influenced by observer bias among healthcare providers and the generally higher severity of conditions in patients attending this department. Therefore, the specific question raised by the reviewer regarding missed diagnosis by the healthcare providers through targeted PITC in the emergency department cannot be addressed.

• *Factors associated with positive HIV test: this analysis appears to be using data from all clients enrolled in the study and not just those targeted for PITC. This is where it gets rather confusing for the reader as the paper on one hands presents yield of targeted PITC with a subgroup selected for PITC, and then factors associated with positive result within a bigger subgroup of n=5028 whose characteristics are not presented in any of the previous tables.*

RE: Thank you for the comment, and we apologise for any confusion caused. During the observation phases, we offered HIV testing to all individuals presenting at the healthcare facility who accepted to participate in the study, driven by two key reasons: i) to identify HIV diagnoses missed by the current targeted PITC algorithm, and ii) to explore factors associated with a positive HIV test beyond those included in the current targeted PITC algorithm, necessitating the inclusion of not just patients referred for targeted PITC to minimise potential selection bias. We have detailed this approach in the Methods section at line 192. Additionally, it is important to note that the subgroup of 5,014, used in the secondary analysis of factors associated with a positive HIV test, was derived from the total number of individuals recruited during both observation phases (n=7,102). Out of these, 2,074 were not tested for HIV due to either a previously known HIV diagnosis or refusal to test, and 14 had undetermined HIV test result, as illustrated in Figure 2 and clarified in the Results section at line 331. This led to a final count of 5,014 participants being included in the analysis of factors associated with a positive HIV test result. The characteristics of this cohort are displayed in the "Total" column of Table 3.

• *Results, page 45, line 361: the coauthors state targeted PITC would have identified 72% of all persons with a positive result (306 of 509), but it is not clear if most of these were*

Fundación número 2634. Registro de Fundaciones Generalitat de Catalunya. NIF: G-65341695

*picked up by the healthcare workers who referred them for PITC despite not meeting the algorithm criteria?*

RE: Thank you for the comment. As illustrated in Figure 2, out of the 509 individuals with a positive HIV test, 364 were eligible for HIV testing according to the current targeted PITC algorithm, with healthcare providers referring 360 of these for testing. Of the 145 individuals with a positive HIV test but not eligible under the current targeted PITC criteria, 116 were also referred for testing. This reflects a practice during the observation phases that leaned more towards universal rather than targeted PITC, likely influenced by the Hawthorne effect. However, it is important to note that at line 366, we aimed to describe the number of HIV diagnoses missed by the current targeted PITC algorithm eligibility, regardless of healthcare provider referrals. We have provided additional clarification at this line. The performance of targeted PITC as executed by healthcare providers, measured as HIV test positivity and being the primary outcome of this study, has been thoroughly presented earlier in the section of HIV positivity yield in the Results at line 301.

*Figure 2 is very unclear - impossible to read.*

RE: Thank you for the comment, and we apologise for any confusion caused. As previously mentioned, in response to feedback during the first revision, we improved the resolution of Figure 2, an extensive flow diagram. Unfortunately, despite our efforts to enhance its resolution, it seems the clarity remained compromised in the PDF document generated by the submission process. We believe that the figure can be downloaded from the submission system in its original format for clearer viewing. To aid the review process, and as the reviewer must have already noted, we have attached Figure 2 to this letter.

----------------------------------------------------------------------------------------------------

Thank you for considering this article for publication in your journal. I look forward to hearing from you soon.
Please accept my highest assurances.

Yours sincerely,

Fundación número 2634. Registro de Fundaciones Generalitat de Catalunya. NIF: G-65341695

Anna Saura Lázaro

Anna Saura

ISGlobal, Hospital Clínic - Universitat de Barcelona, C/ Rosselló, 132, 5th 2nd. 08036. Barcelona, Spain. Phone: (+34) 652413532. Email: anna.saura@isglobal.org

Fundación número 2634. Registro de Fundaciones Generalitat de Catalunya. NIF: G-65341695
